# Effect of Timing Fertilizer Application on leaf yield and quality of tobacco

**DOI:** 10.1016/j.heliyon.2023.e19670

**Published:** 2023-08-30

**Authors:** Jacob Bulenga Lisuma, Johnson Mashambo Semoka, Abraham Furahini Mbwambo

**Affiliations:** a Research Department; Tobacco Research Institute of Tanzania (TORITA), P.O Box 431, 45120 Tumbi, Tabora, Tanzania; b Soil and Geological Sciences Department; Sokoine University of Agriculture (SUA), P.O Box 3008 Morogoro, Tanzania

## 1 Introduction

Tobacco (*Nicotiana tabacum* L.) is an important cash crop to most farmers in Western and Central Zones of Tanzania [1]. However, the crop yield is adversely affected by low soil fertility [2]. The nutritional requirement for the crop to provide potential yield-is high and requires huge amount of nitrogen (N), phosphorous (P), and potassium (K) for proper growth and development of roots [[3], [4], [5], [6], [7]]. After being transplanted in fields, tobacco seedlings usually require a basal application of N_10_P_18_K_24_ fertilizer [8]. In Tanzania, this basal application is carried out 7 days after transplanting (DAT), followed by a top-dressing of CAN (27% N) at 21 DAT [9].

Database from Tanzania Tobacco Board (TTB) shows that tobacco leaf yield for the past decade ranged between 50,000 and 90,000 tons depending on registered number of tobacco growers [10]. Based on these data, the application of N_10_P_18_K_24_ fertilizers had not significantly improved the tobacco leaf yields per unit area in the country. Instead, tobacco leaf improvements are usually noticed after top-dressing application [11]. This indicates that the application of N_10_P_18_K_24_ does not have significant contribution to leaf yield and quality, probably due to limited root growth and development to absorb nutrients substantially.

Larsen et al. [12] and Cochavi et al. [13] demonstrated that the interface between functional roots and soil is the site where water and nutrient absorption is facilitated. Other researchers showed that root length density and hairs on root surface facilitate anchoring of the plant in the soil [[14], [15], [16]]. Stemming on these observations, application of fertilizers while plant roots are not anchored to the soil results in loss of applied nutrients, particularly N, as roots do not yet have the capacity to absorb nutrients efficiently [17]. Hence different strategies including split applications, coating, pelleting and slow release fertilizers, may be applied to improve N use [[18], [19], [20]].

Although the role of fertilizer application in tobacco is well known it is neither linked to the establishment of roots after transplanting nor appropriate timing of N_10_P_18_K_24_ application for substantial uptake of nutrients by the roots. In addition, there are different perceptions by farmers in Tanzania on quality performance of fertilizers to their crops [21]. Understanding the appropriate period for N_10_P_18_K_24_/CAN application by linking it with establishment of roots is essential for efficient nutrient uptake in poor sandy soils as this will impact on the increase in tobacco leaf yield and quality.

The current study aimed at determining appropriate time for fertilizer application to achieve efficient uptake by tobacco roots.

## 2 Materials and methods

### 2.1 Site description and experimentation

Field experiments were conducted at Tobacco Research Institute of Tanzania (TORITA) in Tumbi, Tabora during two cropping seasons, i.e 2020-2021 and 2021–2022. Tumbi site is located at 05° 03′ 44.4″ S and 032° 40′ 07.4″ E with an altitude of 1151.m above sea level. The site was selected from a virgin area where tobacco has not been planted for over fifteen (15) years. The average temperature and rainfall, in the area were 27 °C and 950 mm for 2020–2021 and 27.3 °C and 990 mm for 2021–2022 cropping season, respectively.

Experiments were laid out in a randomized complete block design with three replications and treatments as indicated in Table 1.

**Table 1:** Fertilizer treatments applied to tobacco seedlings at 7, 14, 21, and 28 DAT.

Treatment label	Rate of basal fertilizer (g plant^−1^)	N_10_P_18_K_24_ application time (DAT)	Rate of top-dressing fertilizer (g plant^−1^)	Top-dressing application time (DAT)
T1	0 (N_0_P_0_K_0_)	–	0 (CAN 27%)	–
T2	30 (N_10_P_18_K_24_)	7	8 (CAN 27%)	21
T3	15 (N_10_P_18_K_24_)	14	8 (CAN 27%)	28
T4	15 (N_10_P_18_K_24_)	14	–	–
T5	10 (N_10_P_18_K_24_)	14	20 (N_10_P_18_K_24_)	28
T6	20 (N_10_P_18_K_24_)	14	10 (N_10_P_18_K_24_)	28
T7	30 (N_10_P_18_K_24_)	14	8 (CAN 27%)	28

Tobacco seed (K326) sourced from TORITA were sown in a seedbed of 1.5 m width and 20 m length. After 60 days of germination and nurturing, seedlings were transplanted on ridges spaced 1.2 m with intra-row spacing of 0.5 m in experimental plots of 6 m × 6 m and inter-plot spacing of 1 m (Table 1). Five ridges were set aside to study root growth and development. The first ridge was used to study the length of the roots horizontally, vertically and to count the number of lateral roots and fibrous roots at transplanting time. Similar assessments were done after 7, 14, 21 and 28 days.

In brief, control (T1) seedlings were not fertilized. Seedlings in standard treatment (T2) received 30 g plant^−1^ of N_10_P_18_K_24_ at 7 DAT and top-dressed with 8 g plant^−1^ of CAN (27% N) at 21DAT (Table 1). Other treatments were as follows: seedlings in T3 received 15g plant^−1^ of N_10_P_18_K_24_ at 14 DAT and top-dressed with 8g plant^−1^ of CAN 27% N at 28 DAT, treatment inT4, was N_10_P_18_K_24_ at 15g plant^−1^ at 14 DAT, treatment T5 involved N_10_P_18_K_24_ applied in two splits of 10g plant^−1^ at 14 DAT and 20g plant^−1^ at 28 DAT, seedlings in treatment T6 also received the two first splits of N_10_P_18_K_24_ at 20g plant^−1^ and 10g plant^−1^ on 14th and 28th DAT, and treatment (T7) where seedlings were given 30g plant^−1^ of N_10_P_18_K_24_ at 14 DAT, and top-dressed with 8g of CAN plant^−1^ at 28 DAT.

Basal N_10_P_18_K_24_ applications in the above treatments were done at 7 and 14 DAT as follows: for T2 used 50 kg N ha^−1^; 90 kg P ha^−1^; 120 kg K ha^−1^, T3 used 25 kg N ha^−1^; 45 kg P ha^−1^; 60 kg K ha^−1^, while T7 used 50 kg N ha^−1^; 90 kg P ha^−1^; 120 kg K ha^−1^. The applications were followed by CAN (27% N) at 21 and 28 DAT for T2, T3 and T7 (34 kg N ha^−1^) (Table 1). For each cropping season, harvesting of tobacco leaves was from the beginning of January to the end of April.

### 2.2 Data collection

#### 2.2.1 Determination of soil fertility before tobacco transplanting

Soil samples were collected in a zig zag pattern from each experimental plot to a depth of 0–30 cm. Eventually, a composite soil sample was collected out of 5 subsamples. This sampling was done to 900 m^2^ each year for each cropping season.

Soil properties were determined before setting up trials. These included: soil pH, OC, total N, extractable P, exchangeable K, Ca and Mg, and extractable Mn, S and B. Soil pH was determined using a soil: water ratio of 1:2.5 [22]. Total N was determined by Kjeldahl method, and OC was determined by Walkley and Black Method [23]. Available P was determined by Bray-1 method [22], while exchangeable Ca and Mg in 1 M neutral ammonium acetate (NH_4_OAc) filtrates were measured by atomic absorption spectrophotometer (AAS). The exchangeable K was determined using a flame photometer [22]. Furthermore, S was extracted by calcium monophosphate (Ca(H_2_PO_4_)_2_) and determined by turbidimetric method, while B was determined by hot water-extractable B [22].

#### 2.2.2 Determination of root growth and number of lateral and fibrous roots

To determine the root growth and number of lateral and fibrous roots, tobacco seedlings were planted in a particular location and observed closely. Horizontal and vertical root growth was determined by digging using a shovel and lifting the seedlings slowly. A measuring tape was used to measure the root length and depth. Subsequently, the uprooted seedlings were slowly immersed in water to dislodge adhering soil particles, dried for a week, and then counted for lateral and fibrous roots at 7th, 14th, 21st and 28th DAT.

#### 2.2.3 Plant leaf length, width and area determination

Five plants per plot were selected to determine plant leaf length and width using a measuring tape. These parameters were then used to calculate leaf area as described by Yang et al. [24] indicated in the formula below. Three mature leaves from the bottom, middle, and top positions of the plants were measured and a sum average was recorded per plot. Leaf length and width were measured using a measuring tape. A correction factor of 0.6345 was multiplied to the obtained figures to get leaf area [24].

$$A_{s}=\sum_{i=1}^{n}\left(L_{i}\;x\;W_{i}\;x\;0.6345\right)$$

Where, A_s_ = flue cured tobacco leaf area per plant.

n = flue cured tobacco leaf number

$L_{i}$ = largest leaf length

$W_{i}$ = maximum leaf width

#### 2.2.4 Leaf sampling and plant leaf harvesting

A mature middle leaf in each tobacco plant was sampled such that the border rows and the first two plants at the edges of the inner rows were excluded. The leaves were sampled from two plants of the second inner row in each plot. Therefore, a total of 42 plants were sampled in the experimental site. The sampled leaves were dried in an oven at 65 °C at a constant weight, then chopped and sieved through a 0.5 mm wire mesh. With exception to N, which was determined by wet digestion with concentrated H_2_SO_4_, other nutrients (P, K, Ca and B) were determined by dry ashing extraction procedures [22]. Agronomic techniques such as weeding, earthing-up, removal of suckers and field observation of plant growth were made throughout the experimental period.

Ripened plant leaves were harvested weekly from each experimental plot and weighed using a digital scale. Harvested tobacco leaves were tied to a stick and loaded in a curing barn for about 7 days to dry. Dried leaves were offloaded from the curing barn, weighed, graded and allocated price through a registered Classifier from TTB. Grade index was calculated based on the weight of sold tobacco multiplied by the price allocation and divided by the overall tobacco weight.

### 2.3 Statistical analyses

Statistical analyses were done using STATISTICA 8th Edition, StatSoft, Inc, Tulsa, OK 74104 USA one way analysis of variance (ANOVA). The significant means were compared using Fisher's least significant difference at *p* = 0.05.

## 3 Results

### 3.1 Chemical properties of soils

The chemical properties of Tumbi soil are presented in Table 2. The textural class of the study site was sandy soil with silt 0.92%, clay 7.4% and sand 91.68%. The soil pH was very acidic (5.25), OC was medium (1.93%), total soil N was low (0.15%), soil available (P) was deficient (3.16 mg kg^−1^), exchangeable soil K was deficient (0.05 mg kg^−1^), while exchangeable Ca and Mg were low. Soil Mn was medium (10.73 mg kg^−1^, soil S was high (79.65 mg kg^−1^), and low soil B (0.03 mg kg^−1^). All ratings of the chemical properties were based on Landon [25].

**Table 2:** Values of soil quality parameters determined in the study area.

Soil pH	OC (%)	Total N (%)	P (mg kg^−1^)	K (mg kg^−1^	Ca cmol(+) kg^−1^	Mg cmol(+) kg^−1^	Mn (mg kg^−1^)	S (mg kg^−1^)	B (mg kg^−1^)
5.25	1.93	0.15	3.16	0.05	1.34	0.30	10.73	79.65	0.03

### 3.2 Determination of root growth

**Fig. 1:**
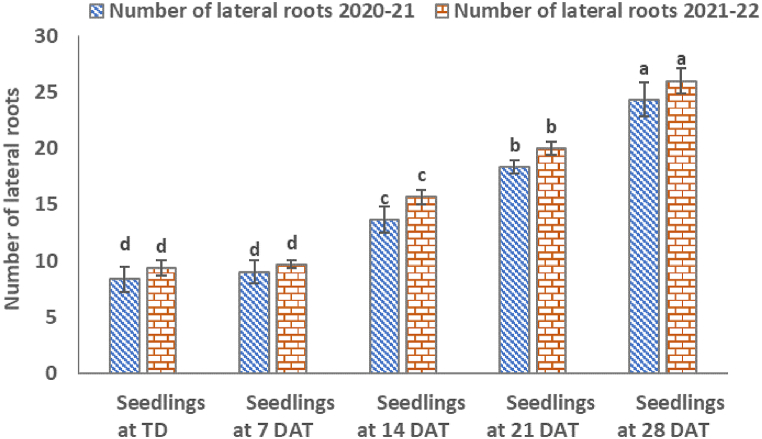
Number of lateral roots counted on transplanting day and at intervals of 7 days.

**Fig. 2:**
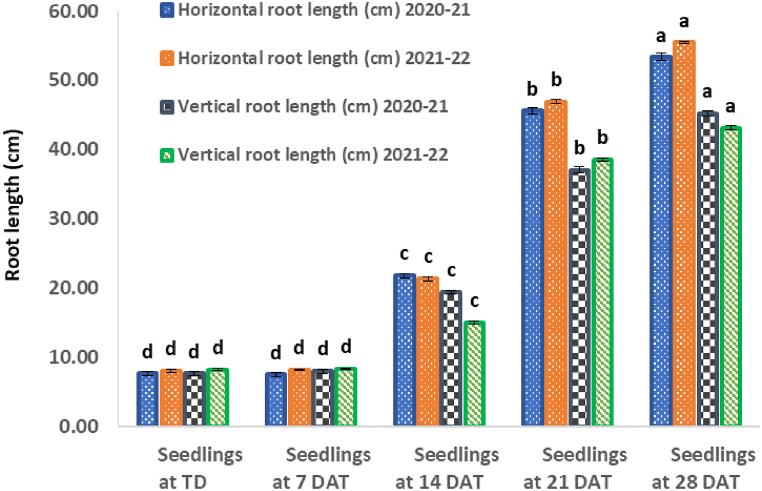
Horizontal and vertical root length counted at intervals of 7 days.

**Fig. 3:**
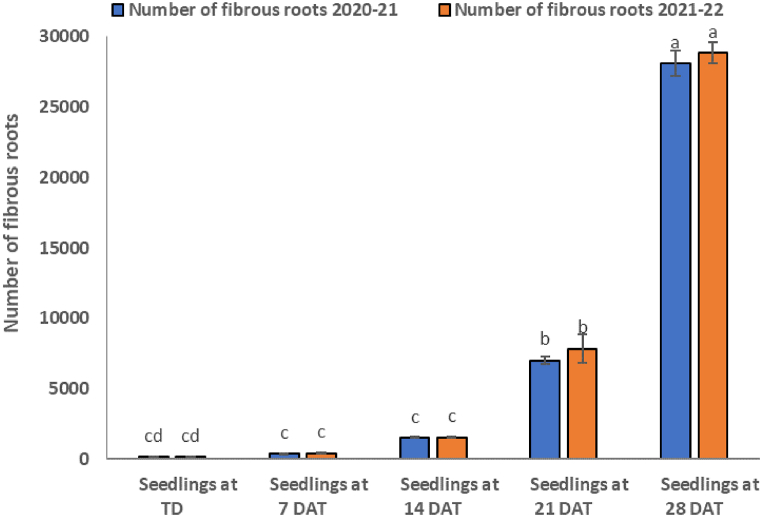
Number of fibrous roots counted at intervals of 7 days.

On the transplanting day (TD) and 7 DAT, the number of lateral and lengths of horizontal and vertical roots did not differ significantly (*P ≤*0.001) during both cropping seasons (Fig. 1, Fig. 2). However, the number of fibrous roots differed slightly at 7 DAT compared to those at transplanting time for both cropping seasons (Fig. 3).

According to Fig. 1, the numbers of lateral roots at TD were 8 and 9 for 2020–2021 and 2021–2022 cropping seasons, respectively. On 7th DAT, the numbers were 9 and 10 for the two seasons (Fig. 1). Horizontal root lengths for the two seasons on TD were 7.67 and 8.07 cm, respectively.These parameters did not differ significantly at 7 DAT, which were 7.50 and 8.17 cm long, respectively. Vertical root lengths followed similar trends to horizontal lengths. They were 7.67 and 8.23 cm for the two seasons, respectively. The lengths 8.00 and 8.23 cm at 7 DAT (Fig. 2). Counted fibrous roots on TD were 175 and 183. They increased slightly at 7 DAT to 399 and 429 respectively (Fig. 3).

**Fig. .4:**
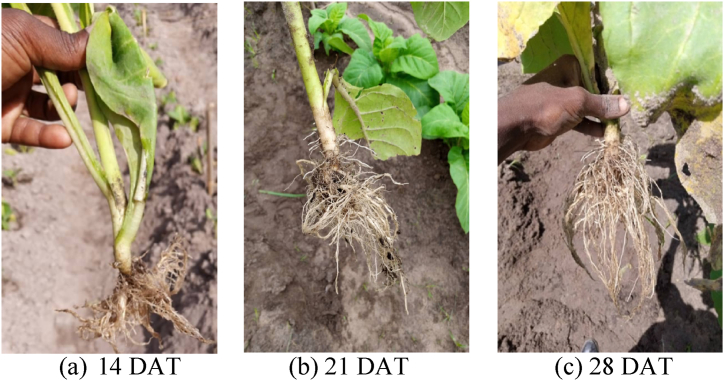
Root growth patterns observed at 14, 21 and 28 days after transplanting.

The number and length of roots for tobacco seedlings at 14, 21 and 28 DAT differed significantly (Fig. 4; a, b and c). The horizontal lengths were 21.80; 21.37, 45.57; 46.87; 53.33; 55.43 cm for the 2020-21 and 2021-22 cropping seasons, respectively, and the vertical root lengths were 19.40; 15.03, 37.07; 38.53, 45.23; 43.13 cm for the same cropping seasons at 14, 21 and 28 DAT tobacco seedlings. The numbers of lateral roots were 14; 16, 18; 20 and 24; 26 at14, 21 and 28 DAT in the 2020-21 and 2021-22 cropping seasons, respectively. In the same period, the counted numbers of fibrous roots increased tremendously to 1546and 1561, 7033and 7847, and 28132and 28878 during both cropping seasons at 14, 21 and 28 DAT.

### 3.3 Effects of different fertilizer application time and rates on leaf area, yields and grade index

Leaf area, green leaf yields, dry leaf yields and grade index during the 2020–2021 and 2021–2022 cropping seasons differed significantly (*P* ≤ 0.001) across treatments (Table 3, Table 5). Treatment T5, for both cropping seasons had significantly (*P* ≤ 0.001) highest leaf area (992.16; 1019.97 cm^2^), green leaf yield (15856.47; 15867.99 kg ha^−1^), dry leaf yield (2085.18; 2694.82 kg ha^−1^) and grade index (0.96; 0.96) followed by T7 (Table 3, Table 4). Treatment T6, had superior results to the control treatment (T2), which had leaf areas of 863.34 and 889.17 cm^2^, green leaf yields of 12685.17 and 12696.69 kg ha^−1^, dry leaf yields of 2355.08 and 2364.72 kg ha^−1^ and grade indices of 0.79 and 0.85 for the two cropping seasons, respectively. Lowest yields were observed in control (T1), and improved slightly for T4.

**Table 3:** Fertilizer effects on leaf area, green leaf yield, dry leaf yield and grade indices of tobacco during 2020–2021 cropping season.

	Treatments	Leaf area (cm^2^)	Green leaf yield (kg ha^−1^)	Dry leaf yield (kg ha^−1^)	Grade Index
1	Control	569.63 ± 5.13 f	6541.66 ± 0.54 g	1252.49 ± 0.89 g	0.22 ± 0.01 e
2	N_10_P_18_K_24_ 30g 7 DAT + CAN 27% 8g plant^−1^ at 21DAT	863.34 ± 3.77 c	12685.17 ± 0.54 d	2355.08 ± 0.89 d	0.79 ± 0.02 b
3	N_10_P_18_K_24_ 15g at 14 DAT + CAN 27% 8g plant^−1^ at 28DAT	788.76 ± 3.63 d	12115.81 ± 0.55 e	2090.54 ± 0.89 e	0.56 ± 0.01 c
4	N_10_P_18_K_24_ 15g at 14 DAT	737.53 ± 3.52 e	10632.62 ± 0.55 f	1987.86 ± 0.89 f	0.37 ± 0.01 d
5	N_10_P_18_K_24_ 10g at 14 DAT + N_10_P_18_K_24_ 20g plant^−1^ at 28DAT	992.16 ± 4.07 a	15856.47 ± 0.54 a	2685.18 ± 0.89 a	0.96 ± 0.01 a
6	N_10_P_18_K_24_ 20g at 14 DAT + N_10_P_18_K_24_ 10g plant^−1^ at 28DAT	867.09 ± 3.82 c	14666.66 ± 0.54 c	2617.58 ± 0.89 c	0.80 ± 0.01 b
7	N_10_P_18_K_24_ 30g at 14 DAT + CAN 27% 8g plant^−1^ at 28DAT	938.83 ± 3.96 b	15504.62 ± 0.55 b	2622.67 ± 0.89 b	0.93 ± 0.01 a
	F-statistics	1220.20***	3.55x107***	324834***	164.73***

**Key**: Means in the same category of evaluated interface sharing similar letter(s) do not differ significantly based on their respective Standard error (SE) at 5% error rate. Values presented are means ± SE(Standard error of means); *** means significant at *P* < 0.001; DAT = days after transplanting tobacco seedlings.

**Table 4:** Fertilizer effects on leaf area, green leaf yield, dry leaf yield and grade indices of tobacco during the 2021–2022 cropping season.

	Treatments	Leaf area (cm^2^)	Green leaf yield (kg ha^−1^)	Dry leaf yield (kg ha^−1^)	Grade Index
1	Control	598.29 ± 10.26 f	6553.18 ± 0.81 g	1262.13 ± 1.74 f	0.29 ± 0.01 e
2	N_10_P_18_K_24_ 30g 7 DAT + CAN 27% 8g plant^−1^ at 21DAT	889.17 ± 11.64 c	12696.69 ± 0.81 d	2364.72 ± 1.74 c	0.85 ± 0.01 b
3	N_10_P_18_K_24_ 15g at 14 DAT + CAN 27% 8g plant^−1^ at 28DAT	813.65 ± 11.17 d	12127.33 ± 0.81 e	2100.18 ± 1.74 d	0.60 ± 0.01 c
4	N_10_P_18_K_24_ 15g at 14 DAT	761.63 ± 10.84 e	10644.14 ± 0.81 f	1997.50 ± 1.24 e	0.41 ± 0.01 d
5	N_10_P_18_K_24_ 10g at 14 DAT + N_10_P_18_K_24_ 20g plant^−1^ at 28DAT	1019.97 ± 12.53 a	15867.99 ± 0.81 a	2694.82 ± 1.74 a	0.96 ± 0.02 a
6	N_10_P_18_K_24_ 20g at 14 DAT + N_10_P_18_K_24_ 10g plant^−1^ at 28DAT	893.25 ± 11.77 c	14678.18 ± 0.81 c	2627.22 ± 1.74 b	0.93 ± 0.00 ab
7	N_10_P_18_K_24_ 30g at 14 DAT + CAN 27% 8g plant^−1^ at 28DAT	965.93 ± 12.22 b	15516.14 ± 0.81 b	2632.31 ± 1.78 b	0.96 ± 0.06 a
	F-statistics	148.48***	1.61x107***	85005***	98.76***

**Key**: Means in the same category of evaluated interface sharing similar letter(s) do not differ significantly based on their respective Standard error (SE) at 5% error rate. Values presented are means ± SE_x̅_ (Standard error of means); *** means significant at *P* < 0.001; DAT = days after transplanting tobacco seedlings.

**Table 5:** Nutrient concentrations in tobacco leaf during the 2020–2021 cropping season.

	Treatments	B (mg kg^−1^)	N (%)	P (%)	K (%)	Ca (%)
1	Control	13.95 ± 0.04 g	1.51 ± 0.01 e	0.12 ± 0.01 f	1.60 ± 0.01 e	1.91 ± 0.02 f
2	N_10_P_18_K_24_ 30g 7 DAT + CAN 27% 8g plant^−1^ at 21DAT	15.13 ± 0.04 d	1.87 ± 0.01 c	0.16 ± 0.01 e	2.72 ± 0.14 c	2.23 ± 0.01 cd
3	N_10_P_18_K_24_ 15g at 14 DAT + CAN 27% 8g plant^−1^ at 28DAT	14.72 ± 0.04 e	1.69 ± 0.01 d	0.24 ± 0.01 c	2.19 ± 0.03 d	2.35 ± 0.01 b
4	N_10_P_18_K_24_ 15g at 14 DAT	14.32 ± 0.01 f	1.52 ± 0.01 e	0.20 ± 0.01 d	2.15 ± 0.01 d	2.06 ± 0.01 e
5	N_10_P_18_K_24_ 10g at 14 DAT + N_10_P_18_K_24_ 20g plant^−1^ at 28DAT	18.07 ± 0.06 a	3.51 ± 0.01 a	0.71 ± 0.01 a	3.71 ± 0.12 a	2.26 ± 0.01 c
6	N_10_P_18_K_24_ 20g at 14 DAT + N_10_P_18_K_24_ 10g plant^−1^ at 28DAT	15.34 ± 0.02 c	3.38 ± 0.03 b	0.63 ± 0.01 b	2.94 ± 0.01 c	2.18 ± 0.02 d
7	N_10_P_18_K_24_ 30g at 14 DAT + CAN 27% 8g plant^−1^ at 28DAT	17.28 ± 0.02 b	3.41 ± 0.03 b	0.66 ± 0.01 b	3.40 ± 0.01 b	3.46 ± 0.02 a
	F-statistics	1522***	2453.10***	712.98***	102.13***	967.40***

**Key**: Means in the same category of evaluated interface sharing similar letter(s) do not differ significantly based on their respective Standard error (SE) at 5% error rate. Values presented are means ± SE_x̅_ (Standard error of means); *** means significant at *P* < 0.001; DAT = days after transplanting tobacco seedlings.

### 3.4 Effects of time of fertilizer application on nutrient contents of tobacco leaves

Leaf nutrient concentrations in tobacco for the cropping seasons (2020–2021 and 2021–2022) are presented in Table 5, Table 6. In both cropping seasons, significantly (*P* ≤ 0.001) higher tobacco leaf nutrient concentrations as an effect of application of different fertilizer rates and time were observed in T5 for B (18.07; 18.74 mg kg^−1^), N (3.51; 4.21%), P (0.71; 0.85%) and K (3.71; 3.91%), followed by treatment T7, which had B (17.28; 17.37 mg kg^−1^), N (3.41; 3.50%), P (0.66; 0.74%), K (3.40; 3.56%), but had a significant (*P* ≤ 0.001) higher leaf nutrient Ca (3.46; 3.53%) than T5 during the cropping seasons, respectively. The lowest leaf nutrient concentrations were recorded in the control (T1). Furthermore, these results show that, application of fertilizer at 14 DAT influenced improvement of leaf nutrient concentrations at a decreasing order of T5>T7>T6 > T3 > T4.

**Table 6:** Nutrient concentrations in tobacco leaf during the 2021–2022 cropping season.

	Treatments	B (mg kg^−1^)	N (%)	P (%)	K (%)	Ca (%)
1	Control	14.30 ± 0.02 g	1.57 ± 0.01 f	0.15 ± 0.01 d	1.74 ± 0.01 f	1.80 ± 0.03 f
2	N_10_P_18_K_24_ 30g 7 DAT + CAN 27% 8g plant^−1^ at 21DAT	15.85 ± 0.01 c	1.89 ± 0.01 d	0.16 ± 0.01 d	2.65 ± 0.01 d	2.30 ± 0.01 d
3	N_10_P_18_K_24_ 15g at 14 DAT + CAN 27% 8g plant^−1^ at 28DAT	15.12 ± 0.01 e	1.70 ± 0.01 e	0.32 ± 0.01 c	2.21 ± 0.01 e	2.43 ± 0.01 bc
4	N_10_P_18_K_24_ 15g at 14 DAT	14.62 ± 0.01 f	1.57 ± 0.00 f	0.29 ± 0.01 c	2.17 ± 0.01 e	2.11 ± 0.01 e
5	N_10_P_18_K_24_ 10g at 14 DAT + N_10_P_18_K_24_ 20g plant^−1^ at 28DAT	18.74 ± 0.01 a	4.21 ± 0.01 a	0.85 ± 0.01 a	3.91 ± 0.01 a	2.46 ± 0.01 b
6	N_10_P_18_K_24_ 20g at 14 DAT + N_10_P_18_K_24_ 10g plant^−1^ at 28DAT	15.54 ± 0.01 d	3.47 ± 0.01 c	0.72 ± 0.01 b	3.02 ± 0.02 c	2.41 ± 0.01 c
7	N_10_P_18_K_24_ 30g at 14 DAT + CAN 27% 8g plant^−1^ at 28DAT	17.37 ± 0.01 b	3.50 ± 0.01 b	0.74 ± 0.02 b	3.56 ± 0.01 b	3.53 ± 0.02 a
	F-statistics	13876***	18083.1***	605.47***	2849.2***	1016.10***

**Key**: Means in the same category of evaluated interface sharing similar letter(s) do not differ significantly based on their respective Standard error (SE) at 5% error rate.

Values presented are means ± SE_x̅_ (Standard error means); *** means significant at *P* < 0.001; DAT = days after transplanting tobacco seedlings.

## 4 Discussion

### 4.1 Influence of time of exposure on the establishment of lateral and fibrous roots

Results in this study have revealed that at 7 DAT (Table 1), the number and length of lateral-, horizontal- and vertical roots did not increase significantly (*P ≤*0.001) during the 2020–2021 and 2021–2022 cropping seasons (Fig. 1, Fig. 2). This, indicates that transplanted tobacco seedlings needed more time for the roots to acclimatize to the new growing soil media. However, at 7 DAT only fibrous roots showed a sign of regrowth (Fig. 3). For both cropping seasons, fibrous roots regrowth increased by 128–134%, indicating that tobacco fibrous roots initiation begins within 7 DAT.

Two weeks after transplanting the seedlings, a significant (*P ≤*0.001) increase in the number of lateral roots by 55–60% was observed in both cropping seasons (Fig. 4a). These roots are vital in anchoring the plants in soils. Subsequently, a significant (*P ≤*0.001) increase in horizontal root length by 165–184% was exhibited. The latter strengthened seedling establishment in the soil and enhanced increase in vertical root length by 83–153% which improved further the anchoring of plants in the soil. Indeed, this development led to a significant (*P ≤*0.001) increase in the number of fibrous roots by 753–783% and improved the nutrient and water acquisition.

There was a further increase in the number of lateral roots by 100% at 21 DAT that led to significant (*P ≤*0.001) increase in both horizontal (481–494%) and vertical (368–383%) root length (Fig. 4b). These trends were associated with significant (*P ≤*0.001) increases in fibrous roots by 3919–4188% which in turn increased the surface area for gas exchange, nutrient and water absorption as reported by other researchers [26].

At 28 DAT (Fig. 4c)root lengths increased by 160–167% (lateral), 587–595% (horizontal), 424–490% (vertical), and massively by 15680–15975% (fibrous). This trend shows that lateral tobacco roots increased by 50% every week to support the anchorage of tobacco seedlings. Previous studies have shown that the increase in lateral roots is a prelude to increase in fibrous, horizontal and vertical root length [[26], [27], [28]]. These results indicate that at 28 DAT, roots could be approaching maximum growth or facing compression challenges in growing deeper into the soil [29].

### 4.2 Effect of time exposure for plant growth and application of fertilizer on leaf area, yield and grade

Tobacco planted in both cropping seasons (Table 2) had significant (*P ≤*0.001) higher leaf area, green, dry leaf yield and grade index in T5 than any other treatments (Table 3, Table 4). This could be due to the application of N_10_P_18_K_24_ fertilizer in two splits that provided high nutrient levels for a relatively long period. Basically, the increase in rooting surface observed in this study created an environment for efficient utilization of applied nutrients at the lowest rate since roots were developing fast. This is in consistent with previous findings [27].

The second split, for treatment T5 increased further the number of lateral roots, which in turn increased nutrient and water absorption. More nutrients' absorption increased leaf area, green and dry leaf yield (Table 4, Table 5). These traits contributed to the quality of tobacco leaf as the measured leaf nutrient (B, N, P, K and Ca) concentrations (Table 5, Table 6) were significantly higher than any other treatments and had sufficient leaf nutrient concentrations as described by Bryson and Mills [30]. The root surface area at 14 DAT could not absorb significant nutrients applied at the higher N_10_P_18_K_24_ rate (Fig. 1, Fig. 2, Fig. 3). In addition, the applied N in NPK fertilizer might have delayed tobacco growth [31]. Thus, application of nutrients at 28 DAT seems to be appropriate time for high yield and quality.

Furthermore, application of CAN fertilizer was observed to be of low significance as all treatments (T2, T3 and T7) did not produce high yields with good leaf quality than T5. This signifies that Ca supplied by the soil was adequate and the tobacco plants good indices for absorbing Ca since even controls had sufficient Ca levels. Wise application of chemical and organic fertilizers in cropping systems is key to their high use efficiency and maximum benefits [32]. Further research is required for splitting fertilizer application into two splits, as the current study did not have more options to determine the best splitting option.

## 5 Conclusion

The present study has revealed that N_10_P_18_K_24_ application to tobacco plant requires two splits. The first split uses a lowe rate, 10g plant^−1^ of N_10_P_18_K_24_ at 14 DAT, as roots establishment and development are not capable of utilising high rates. The second split, 20g plant^−1^, should be applied at 28 DAT since at this time, lateral roots have increased tremendously in number with massive amount of fibrous roots to absorb nutrients both horizontally and vertically from the soil. However, in order to evaluate the best splitting option, further research is required as the current study had only two splitting treatments. Our findings in this study will help tobacco growers to reduce the cost of production, increase productivity per unit area, and improve their livelihood.

## Author contribution statement

Jacob Bulenga Lisuma, PhD: Conceived and designed the experiments; Performed the experiments; Analyzed and interpreted the data; Contributed reagents, materials, analysis tools or data; Wrote the paper.

Johnson Mashambo Semoka: Analyzed and interpreted the data; Wrote the paper.

Abraham Furahini Mbwambo: Performed the experiments; Wrote the paper.

## Data availability statement

Data included in article/supp. material/referenced in article.

## Additional information

No additional information is available for this paper.

## Declaration of competing interest

The authors declare that they have no known competing financial interests or personal relationships that could have appeared to influence the work reported in this paper.

## Abbreviations

CAN: Calcium ammonium nitrate
DAT: Days after transplanting
NPK: Nitrogen phosphorous potassium
T: Treatment
TD: Transplanting Day
